# Reducing bias in open-label trials where blinded outcome assessment is not feasible: strategies from two randomised trials

**DOI:** 10.1186/1745-6215-15-456

**Published:** 2014-11-21

**Authors:** Brennan C Kahan, Suzie Cro, Caroline J Doré, Daniel J Bratton, Sunita Rehal, Nick A Maskell, Najib Rahman, Vipul Jairath

**Affiliations:** Pragmatic Clinical Trials Unit, Queen Mary University of London, E1 2AB London, UK; MRC Clinical Trials Unit at UCL, London, WC2B 6NH UK; Comprehensive Clinical Trials Unit at UCL, London, WC1E 6BT UK; Academic Respiratory Unit, University of Bristol, Bristol, BS10 5NB UK; Oxford Respiratory Trials Unit, Oxford Centre for Respiratory Medicine, Oxford, OX3 7LJ UK; Nuffield Department of Medicine, University of Oxford, Oxford, OX3 9DU UK

**Keywords:** Randomised controlled trial, Open-label, Unmasked, Subjective outcome, Unblinded outcome assessment, Bias

## Abstract

**Background:**

Blinded outcome assessment is recommended in open-label trials to reduce bias, however it is not always feasible. It is therefore important to find other means of reducing bias in these scenarios.

**Methods:**

We describe two randomised trials where blinded outcome assessment was not possible, and discuss the strategies used to reduce the possibility of bias.

**Results:**

TRIGGER was an open-label cluster randomised trial whose primary outcome was further bleeding. Because of the cluster randomisation, all researchers in a hospital were aware of treatment allocation and so could not perform a blinded assessment. A blinded adjudication committee was also not feasible as it was impossible to compile relevant information to send to the committee in a blinded manner. Therefore, the definition of further bleeding was modified to exclude subjective aspects (such as whether symptoms like vomiting blood were severe enough to indicate the outcome had been met), leaving only objective aspects (the presence versus absence of active bleeding in the upper gastrointestinal tract confirmed by an internal examination).

TAPPS was an open-label trial whose primary outcome was whether the patient was referred for a pleural drainage procedure. Allowing a blinded assessor to decide whether to refer the patient for a procedure was not feasible as many clinicians may be reluctant to enrol patients into the trial if they cannot be involved in their care during follow-up. Assessment by an adjudication committee was not possible, as the outcome either occurred or did not. Therefore, the decision pathway for procedure referral was modified. If a chest x-ray indicated that more than a third of the pleural space filled with fluid, the patient could be referred for a procedure; otherwise, the unblinded clinician was required to reach a consensus on referral with a blinded assessor. This process allowed the unblinded clinician to be involved in the patient’s care, while reducing the potential for bias.

**Conclusions:**

When blinded outcome assessment is not possible, it may be useful to modify the outcome definition or method of assessment to reduce the risk of bias.

**Trial registration:**

TRIGGER: ISRCTN85757829. Registered 26 July 2012.

TAPPS: ISRCTN47845793. Registered 28 May 2012.

## Background

The main goal of a randomised controlled trial (RCT) is to ensure that, apart from the intervention, there are no systematic differences between treatment groups under study, thereby ensuring an unbiased estimate of treatment effect. However, randomisation alone does not preclude the possibility of systematic differences. If patients, clinicians, or assessors are aware of treatment assignments, this may influence reporting or measurement of the outcome and introduce bias [1]. For example, enthusiasm for a newer treatment could lead to better outcomes being reported in this group of patients, regardless of any treatment efficacy.

It is therefore recommended that trials be blinded (or masked) as far as possible to limit bias [2, 3]. However, in some cases the nature of the treatments under investigation may not permit blinding. This can be an issue in trials assessing surgical interventions, device trials, or other non-pharmacologic interventions, which are more difficult to blind than traditional drug trials [4]. Many such trials are therefore open-label, where patients, clinicians, and care providers are aware of treatment allocations.

For objective outcomes, such as all-cause mortality, unblinded assessment is unlikely to bias the trial results [5]. However, evidence suggests that treatment effect estimates may be exaggerated for subjective outcomes when outcome assessors are not blinded [6–13]. This bias can often be prevented by ensuring outcome assessors are blind to treatment allocation, for example by using independent clinicians who are not otherwise involved in the trial to assess patients, or using a blinded adjudication committee to determine outcome [14]. However, blinded outcome assessment may not always be feasible due to the nature of the trial (for example, when all researchers in a centre are aware of treatment allocation, or relevant information cannot be sent to a blinded adjudication committee).

Developing methods to reduce bias when blinding is not possible has recently been identified as a research priority following a Delphi process involving all UK registered clinical trials units [15]. This article highlights the strategies that were employed in the design and execution of two multi-centre UK trials to reduce potential for bias where blinded outcome assessment was not feasible, with the aim of providing guidance from ‘real-life’ examples to clinicians and researchers planning studies with similar challenges.

## Methods

### Issues to consider for blinded outcome assessment

The goal of blinded outcome assessment is to ensure that bias is not introduced due to knowledge of the patient’s treatment group. However, blinded outcome assessment alone is not sufficient to prevent bias; it is also necessary to ensure that the information upon which the assessment is based is not subject to bias.

For example, consider a trial where a blinded adjudication committee determines whether an adverse event has occurred based on a description of the event prepared by an unblinded researcher. Knowledge of the patient’s treatment arm may influence how the event summary was prepared by the researcher (for example, under or overstating certain aspects), which could in turn affect the assessment of the adjudication committee.

Therefore, prevention of outcome assessment bias requires two components. The first is that those making the assessment are blinded to which treatment arm the patient is in. The second is that the information upon which the assessment is made cannot have been influenced by knowledge of the treatment assignment.

### When is blinded assessment not possible?

Two common methods of blinded outcome assessment are to have a blinded clinician directly assess the patient to determine the outcome, or to have a blinded adjudication committee assess the outcome based on information provided about the patient. We discuss some scenarios below where these methods of blinded assessment may be difficult or impossible to achieve.

#### Direct assessment by a blinded clinician

Blinded assessment may not be feasible in trials where all clinicians or researchers in a centre are aware of treatment assignment by necessity (for example, in a cluster randomised trial where all patients in a centre receive the same treatment). In some trials, it may be logistically impossible to ensure a blinded clinician is always available to assess the patient; this is a particular issue when the timings of assessment occur randomly, for example if patients need to be assessed when symptoms occur, rather than at fixed time points.

#### Blinded adjudication committee

The use of a blinded adjudication committee may be impractical when the relevant information is not available to send to the committee, or when the information provided to the committee may have been influenced by knowledge of the treatment allocation. For example, a photo or a video may be required for the committee to make an informed assessment; however, the technology may not be routinely available for this. In other cases, the committee may require a narrative of the event or symptoms for their assessment. However, if it is only feasible for an unblinded person to prepare this narrative, this could introduce bias despite the use of a blinded adjudication committee.

#### Outcomes that are not assessed

Some outcomes may be subject to bias due to lack of blinding, but are not formally assessed (making it impossible for a blinded adjudication committee to eliminate bias). An example of this type of outcome is whether a patient received ancillary interventions or supplemental care (that is a co-intervention) during the follow-up period [3]. This outcome is not assessed (as it either occurred or did not), but could still be subject to bias if the clinician who decides whether to perform the co-intervention is aware of the treatment assignment.

#### Ethical approvals

TRIGGER was conducted according to the declaration of Helsinki, and received ethical approval from the Scotland A Research Ethics Committee (Reference 12/SS/0023) and the NRES Committee South Central - Oxford C (Reference 12/SC/0062). Consent was obtained for data collection on all enrolled patients.

TAPPS was conducted according to the declaration of Helsinki, and received ethical approval from the NRES Committee North West - Preston (Reference 12/NW/0467). All patients provided consent to take part in the trial.

## Results

Using two recent UK multi-centred trials as examples, we highlight the methods that were used to reduce the potential for bias where blinded outcome assessment was not feasible.

### TRIGGER

#### Trial overview

TRIGGER (Transfusion in Gastrointestinal Bleeding) was a cluster randomised feasibility trial comparing two different red blood cell transfusion policies (liberal versus restrictive) in patients presenting to emergency departments in UK hospitals with acute upper gastrointestinal bleeding (AUGIB) [16, 17]. AUGIB is a common medical emergency most commonly caused by peptic ulcer disease. Due to the perceived risk of contamination between treatment policies in an individually randomised design, as well as the inherent challenges of implementing a transfusion policy for an emergency medical condition throughout an entire hospital for the whole of the patient journey, TRIGGER was designed as a cluster randomised trial, meaning that all patients within a given hospital received the same transfusion policy.

Due to the nature of the intervention (administering a blood transfusion), and the fact that the intervention was implemented hospital wide, it was necessary for all clinicians and study personnel in a centre to be aware of the randomised transfusion policy. The primary clinical outcome was further bleeding up to 28 days from presentation.

#### Feasibility of blinded assessment

A direct assessment by a blinded clinician was not feasible for TRIGGER, as all clinicians in a hospital were aware of the allocated treatment and so would be unsuitable. Asking clinicians from another hospital to assess outcomes would have been unrealistic, as further bleeding requires assessment when it is suspected. As this could happen at any time, it would be logistically impossible for a clinician from another hospital to always be available to assess patients.

Using a blinded adjudication committee to determine the presence of further bleeding was also not feasible. Whilst it is possible to take photos of the bleeding lesion during an endoscopy (an internal examination using a fibre optic camera), this facility is not always routinely available, particularly when procedures are performed ‘out of hours’ in an emergency setting, which is likely to occur frequently for a condition like AUGIB. Therefore, the adjudication committee would need to base their decision on a composite of clinical parameters (for example, clinical signs, symptoms, blood tests) which may be a poor surrogate marker of further bleeding, or from a review of case notes or descriptive information prepared by an unblinded clinician, in which case the advantages of a blinded assessor are lost.

#### Assessment method

Because blinded assessment is not feasible, further bleeding was determined by an unblinded clinician. In order to limit the potential for bias due to unblinded assessment, the clinically accepted definition of further bleeding was modified to ensure it was as objective as possible, thus reducing the potential impact of unblinded assessment.

Further bleeding was defined as either ongoing bleeding at the end of the first endoscopy, or bleeding that restarted after initially stopping. Unlike in standard practice, where further bleeding is often defined purely on the basis of surrogate markers including a combination of patient symptoms (for example, vomiting blood) and clinical parameters (for example, a sudden drop in haemoglobin (Hb) count), assessment of bleeding in TRIGGER was via inspection of the patient’s upper gastrointestinal tract to determine whether there was an active bleed; this must have been performed using endoscopy, surgery, or radiology. The outcome assessment was therefore based simply on the presence of blood versus absence of blood, which was unlikely to have been affected by whether the assessor was aware of the treatment allocation.

### TAPPS

#### Trial overview

TAPPS was a randomised trial assessing the management of patients with malignant pleural effusions (fluid in the chest cavity), which often leads to breathlessness and chest pain. Patients were randomised to one of two methods of fluid drainage and administration of talc (which aims to stick the lung to the chest wall and prevent further fluid formation): (a) talc slurry pleurodesis, which involves inserting a chest drain into the pleural space, and inserting talc slurry (a suspension of talc in normal saline in a syringe) through the drain; or (b) thoracoscopic talc poudrage, which involves inserting a thoracoscope (small videoscope) into the pleural space, and inserting talc using a dry spray technique.

The two interventions used different medical instruments and different techniques of reaching the pleural space, and so blinding of patients or study personnel was impossible. The primary outcome was whether the patient received a further procedure for pleural drainage within 90 days of randomisation.

#### Choices of assessment method

Clinicians who were not directly involved in the care of a specific patient were unaware of the treatment allocation, and could therefore be used as blinded assessors to determine whether the patient should undergo further intervention, making direct assessment by a blinded assessor feasible. However, this option was not acceptable practically, as a large proportion of clinicians may feel it is unacceptable for treatment decisions for their patients to be made without their input, and may therefore not be willing to take part in the trial, jeopardising recruitment.

A blinded adjudication committee could assess whether a patient *should* have undergone further pleural drainage, rather than whether they *did* undergo a further procedure. This could be based on a combination of clinical parameters (for example, results from a chest x-ray) and a description of the patient’s symptoms prepared by a blinded clinician. However, an adjudicated outcome of whether the patient should have undergone further pleural drainage could be regarded as a less pragmatic outcome, and thus the treatment effect from such an outcome may not reflect what is likely to occur in usual practice [18]. Additionally, this outcome could be problematic in scenarios where the patient is referred for further drainage when the adjudication committee does not feel it is warranted. This is because the further drainage effectively prevents the patient from developing symptoms which would lead the adjudication committee to recommend further drainage is warranted at a future time point. This is known as a competing-risk [19], and could potentially lead to bias, or a reduction in power.

#### Final decision on assessment method

To limit bias, the decision to refer patients for further drainage was made using a combination of clinical parameters, and blinded and unblinded assessment:

- If a chest x-ray showed that a third or more of the patient’s pleural space was filled with fluid, then the unblinded clinician in charge of patient care could refer the patient for further drainage. There is no need for a blinded assessment in this scenario as it is clear that fluid recurrence has occurred, and further intervention is indicated.
- If less than a third of the pleural space was filled with fluid, then the unblinded clinician in charge of patient care discussed whether further pleural drainage was required (taking care not to unblind them). If the two clinicians disagreed, they discussed the case with the (blinded) chief investigator who made the final decision.

This strategy (a) led to the outcome being based on results from the patient’s chest x-ray when clearly indicated; (b) allowed the unblinded clinician responsible for the patient to be involved in their management; and (c) attempted to mitigate any potential bias from the unblinded clinician by introducing a second, blinded assessor into the process.

## Discussion

Many trial designs do not permit blinding, and are therefore designed as open-label, with patients, clinicians, and other study investigators aware of treatment allocation. Research has suggested that these trials should use blinded outcome assessment to avoid bias in estimated treatment effects [6–10]. Blinded outcome assessment is often achieved by using an independent clinician who directly examines the patient, or by an independent adjudication committee.

However, in some scenarios both of these methods of blinded assessment may be unfeasible. A direct blinded assessment of the patient may be difficult in scenarios where most or all of the staff in a centre are aware of the patient’s treatment, or when assessments occur randomly (for example, when the patient presents due to symptoms). Likewise, the use of a blinded adjudication committee may be complicated in situations where it is difficult to compile information to send to the committee (for example, due to limitations in technology), or when it is necessary for the information to be prepared by unblinded personnel, which could bias results despite the use of a blinded committee. Additionally, some outcomes do not require assessment (for example, whether the patient has received a co-intervention), but may nonetheless be biased by knowledge of the treatment arm.

When blinded outcome assessment is not feasible, it is important to ensure that outcomes are as robust as possible to the lack of blinding. We have demonstrated with both the TAPPS and the TRIGGER trials that outcome definitions or the methods of assessment can be modified to reduce the risk of bias. This has been highlighted as a trial methodology priority, yet there are few examples in the literature to guide clinicians planning trials.

This strategy is particularly important given the shift away from surrogate outcome measures towards outcomes that are directly relevant to patients. For example, previous trials in patients with malignant pleural effusion have often used the amount of fluid on a patient’s chest x-ray as a surrogate for pleurodesis failure [20]. This can be easily assessed by a blinded adjudicator, minimising the risk of bias. However, this outcome is not relevant to patients, as a small amount of fluid on a chest x-ray could still result in severe symptoms, requiring further treatment, and vice versa. Similarly, previous trials in patients with AUGIB have often used a fall in the patient’s Hb level as a surrogate for further bleeding (along with other less objective measures, as described earlier) [21]. Although a drop in the Hb level will be objective (and thus unlikely to be subject to bias), it may be a poor surrogate for actual bleeding episodes. This is because there is sometimes a lag between the development of bleeding and the Hb drop, so patients can experience significant blood loss without a corresponding drop in Hb.

The TAPPS and TRIGGER trials both used patient-relevant outcome measures, which has the benefit of measuring treatment effects which are more relevant to patients. However, the drawback of this approach is that these outcome measure are often more difficult to blind. We have described in this paper the strategies used to reduce this risk of bias, leading to patient-relevant outcomes with minimal risks of bias. In the TRIGGER trial, the outcome definition for further bleeding was modified to exclude the subjective aspects of the definition (for example, clinician assessment of whether patient symptoms were severe enough to indicate bleeding), leaving only the most objective aspects (the presence versus absence of active bleeding visualised in the upper gastrointestinal tract). In the TAPPS trial, the outcome was modified so that the unblinded clinician could only refer the patient for a further procedure when clearly indicated based on the patient’s chest x-ray; otherwise, they needed to reach consensus with a blinded clinician.

Although these strategies should reduce the risk of bias, they are not without limitations. The requirement in TAPPS to use a second (blinded) clinician to assess the patient added extra complexity to the trial, as it required more effort to ensure that clinicians who were not directly involved with the patient were kept blinded, that these clinicians were available to assess the patient when needed, and that this assessment procedure was being followed as specified. The requirement in TRIGGER for all episodes of further bleeding to be confirmed via a procedure (endoscopy, surgery, or radiology) meant that a very small number of bleeding episodes may have been missed in patients who were too unwell to receive a procedure. However, in both cases the benefits of the outcome modification far outweigh the limitations.

Developments in multimedia technology have made blinded assessment for outcomes based on visual inspection (such as further bleeding in TRIGGER) more feasible, as there are often affordable ways of taking high-quality pictures, videos, or other digital recordings, which can be given to an independent adjudicator for assessment. This type of technology has been used in other gastrointestinal trials, for example in the assessment of degree of colonic inflammation in trials of ulcerative colitis [22]. It was felt this type of assessment would not be feasible for TRIGGER due to the difficulties in implementing a new technology across entire hospitals for use in emergency medical settings, particularly in terms of training staff to comply with regulatory and good clinical practice guidelines. However, as trialists become more familiar with the use of multimedia technology in their trials, implementation should become easier, allowing for more objective outcome assessments in certain situations.

## Conclusions

Outcome assessment should be blinded when possible. In scenarios where this is not feasible, it may be beneficial to modify the outcome definition or method of assessment to minimise the subjective elements, to ensure that results are as robust as possible to the lack of blinding.

## Abbreviations

AUGIB: acute upper gastrointestinal bleeding
Hb: haemoglobin
RCT: randomised controlled trial
TAPPS: evaluating the efficacy of thoracoscopy and talc poudrage versus pleurodesis using talc slurry
TRIGGER: Transfusion in Gastrointestinal Bleeding.
